# Account of Diseases in an Eastern District of London, from March 20, to April 20, 1804

**Published:** 1804-05-01

**Authors:** 


### *Account of Diseases in an Eastern District of London, from March 20, to April 20, 1804.*

| | | | |
|---|---|---|---|
| ACUTE DISEASES. | | Fluor Albus | 12 |
| Peripneumonia | 3 | Amenorrhœa | 10 |
| Catarrhus | 4 | Dysuria | 5 |
| Enteritis | 5 | Dyspepsia | 4 |
| Rheumatismus Acutus | 3 | Vertigo | 3 |
| CHRONIC DISEASES. | | Cephalæa | 9 |
| Tussis | 15 | Rheumatismus Chronicus | 12 |
| Tussis cum Dyspnœa | 16 | PUERPERAL DISEASES. | |
| Hæmoptoe | 4 | Dolores post Partum | 6 |
| Phthisis Pulmonalis | 3 | Ephemera | 7 |
| Pleurodynia | 2 | Menorrhagia Lochialis | 5 |
| Hydrothorax | 3 | INFANTILE DISEASES. | |
| Hæmorrhois | 2 | Herpes | 4 |
| Hernia | 1 | Convulsio | 3 |
| Ascites | 4 | Dentitio | 4 |
| Menorrhagia | 7 | Spina Bifida | 1 |

During the last few weeks there has been a considerable variety in the temperature of the atmosphere. At one time, a degree of heat was felt which is unusual at that season of of the year; but since that time the degree of cold has been as remarkably severe. The N. and N. E. winds have prevailed, and their influence has extended over both the vegetable and animal kingdoms. The spring is late, and it is feared that some of the earlier crops of vegetables have been much injured if not entirely destroyed.

The influence of this state of the atmosphere upon the human frame has been very considerable. The severe cold succeeding the unusual degree of warmth has been productive of several acute diseases. Pneumonic complaints have been severe, and affections of the bowels of the acute species have been frequent, and in several instances have terminated fatally.

